# Significance of Autoantibodies in Autoimmune Encephalitis in Relation to Antigen Localization: An Outline of Frequently Reported Autoantibodies with a Non-Systematic Review

**DOI:** 10.3390/ijms21144941

**Published:** 2020-07-13

**Authors:** Keiko Tanaka, Meiko Kawamura, Kenji Sakimura, Nobuo Kato

**Affiliations:** 1 Department of Animal Model Development, Brain Research Institute, Niigata University, 1-757 Asahimachi-dori, Chuoku, Niigata 951-8585, Japan; meiko@bri.niigata-u.ac.jp (M.K.); sakimura@bri.niigata-u.ac.jp (K.S.); 2 Department of Multiple Sclerosis Therapeutics, Fukushima Medical University, School of Medicine, 1 Hikarigaoka, Fukushima 960-1247, Japan; 3 Department of Physiology, Kanazawa Medical University, Ishikawa 920-0293, Japan; kato@kanazawa-med.ac.jp

**Keywords:** autoimmune encephalitis, paraneoplastic, autoantibodies, synapse, cell surface antigen

## Abstract

Autoantibodies related to central nervous system (CNS) diseases propel research on paraneoplastic neurological syndrome (PNS). This syndrome develops autoantibodies in combination with certain neurological syndromes and cancers, such as anti-HuD antibodies in encephalomyelitis with small cell lung cancer and anti-Yo antibodies in cerebellar degeneration with gynecological cancer. These autoantibodies have roles in the diagnosis of neurological diseases and early detection of cancers that are usually occult. Most of these autoantibodies have no pathogenic roles in neuronal dysfunction directly. Instead, antigen-specific cytotoxic T lymphocytes are thought to have direct roles in neuronal damage. The recent discoveries of autoantibodies against neuronal synaptic receptors/channels produced in patients with autoimmune encephalomyelitis have highlighted insights into our understanding of the variable neurological symptoms in this disease. It has also improved our understanding of intractable epilepsy, atypical psychosis, and some demyelinating diseases that are ameliorated with immune therapies. The production and motility of these antibodies through the blood-brain barrier into the CNS remains unknown. Most of these recently identified autoantibodies bind to neuronal and glial cell surface synaptic receptors, potentially altering the synaptic signaling process. The clinical features differ among pathologies based on antibody targets. The investigation of these antibodies provides a deeper understanding of the background of neurological symptoms in addition to novel insights into their basic neuroscience.

## 1. Introduction

Central nervous system (CNS) inflammation associated with autoimmune disorders develops in multiple regional tissues, including the cerebral cortex, cerebral white matter, basal ganglia, brain stem, cerebellum, optic nerve, spinal cord, posterior ganglions, etc. The peripheral nervous system may also be affected. In myasthenia gravis, autoantibodies against acetylcholine receptors (AChRs) functionally block AChRs through the cross-linking and internalization of the receptors at the neuromuscular junction [1,2]. In CNS diseases, several autoantibodies linked to paraneoplastic neurological syndromes (PNS) have been identified, such as the anti-HuD antibodies and anti-Yo antibodies in the 1980s [3,4,5,6]. The discovery of specific autoantibodies has greatly impacted our understanding of progressive neurological disorders. These autoantibodies have excellent roles as diagnostic markers for PNS and early cancer detection. However, they have not been shown to have direct roles in neuronal dysfunction. In 2001, novel autoantibodies against cell surface proteins interacting with voltage-gated potassium channels (VGKC) were reported in two patients presenting with memory loss and seizures. Both of them did not have cancer, and both improved following immunotherapy [7]. In 2004, autoantibodies against aquaporin 4 (AQP4) in patients with neuromyelitis optica (NMO) were reported. These antibodies were detected with cell-based assays (CBA), in which recombinant proteins expressed on the cell membrane of HEK cells preserve their conformational structures [8]. In 2007, another study identified neuronal autoantibodies against the CNS glutamate receptor (NMDAR) in four young women with prominent neuropsychiatric symptoms and ovarian teratoma [9,10]. This approach enabled the identification of several kinds of autoantibodies related to autoimmune encephalomyelitis (AEM).

AEM commonly develops with an acute to subacute time course and is not always associated with systemic inflammatory parameters. AEM frequently causes an increased cell number and/or protein content in the cerebrospinal fluid (CSF) and contributes to abnormal MRI/CT findings in the CNS. However, these findings are not specific to autoimmune diseases. Autoantibodies related to disease phenotypes have been identified, especially those that recognize and target neuronal cell surface synaptic receptors and ion channels in the CNS [11]. The triggers for autoimmunity in CNS tissues are unknown, though some speculate that molecular mimicry mechanisms to prodromal infectious agents and common antigen-presenting tumors in affected tissue may act as triggers. Autoantibodies against synaptic receptors and channels are associated with features of limbic encephalitis, a condition that frequently affects cognition, causing behavioral changes and seizures along with a wide range of other CNS dysfunctions. When antibodies develop against astrocytes or oligodendrocytes, such as AQP4 antibodies or anti-myelin oligodendrocyte glycoprotein (MOG) antibodies, they tend to cause inflammation in the optic nerve and spinal cord structures [8,12]. The associations between autoantibodies and clinical phenotypes help the diagnosis of underlying disorders. However, not all autoantibodies are symptom-specific.

Autoantibodies associated with AEM are divided into three groups based on the localization of their respective antigens within the CNS: (1) intracellular, cytoplasmic, or nuclear; (2) intracellular synaptic sites; and (3) cell surface and membrane-bound. Each group differs in response to treatment, molecular pathogenesis, antibody detection methods, associated conditions, and prognosis. Various autoantibodies related to AEM have been reported; however, not all of them have been proven to be pathogenic (Table 1). Conditions with autoantibody pathogenicity include: (1) the recognition of antigens located on antibody-accessible cell surfaces, whose physiological functions are related to the observed neurological features; (2) the removal of autoantibodies ameliorates symptoms and/or neuroimaging abnormalities; (3) characteristic clinical features are observed in patients positive for the specific antibody; and (4) autoantibodies mostly belong to the immunoglobulin G (IgG) isotype, with their titer correlating with disease activity; (5) if the disease features are replicated in animal models through the use of patient autoantibodies, this would indicate the significant relevance of such autoantibodies in AEM [13]. However, most reported autoantibodies do not satisfy these criteria, especially (5). However, they remain useful for the diagnosis of AEM.

## 2. Autoantibodies Detected in CNS Inflammatory Diseases

### 2.1. Antibodies against Intracellular Antigens

Autoantibodies, which target proteins located in the neuronal cytoplasm or nucleus, usually recognize small peptides and are produced in patients with cancer. These antibodies can be detected in the patients’ sera and are indicative of acutely evolved severe neurological symptoms that usually precede the discovery of the cancer. This group of patients are diagnosed as having a classical form of paraneoplastic neurological syndrome (PNS) [3,4,5,6]. The search for autoantibodies in PNS began in the 1970s with the use of immunohistochemistry and the Western blot. Several diseases were identified in this period. The PNS groups identified throughout this period include (1) limbic encephalitis, encephalomyelitis, or subacute sensory neuronopathy associated with small cell lung cancer and anti-Hu antibodies [15,16]; (2) subacute cerebellar degeneration associated with gynecological or breast cancer and anti-Yo antibodies [17]; (3) opsoclonus-myoclonus syndrome with breast cancer or thymoma and anti-Ri antibodies [18]; (4) encephalomyelitis, optic neuritis, choreatic syndrome, or cerebellar degeneration associated with thymoma or small cell lung cancer and anti-CV2/CRMP5 antibodies [19]; and (5) limbic encephalitis or rhombencephalitis with testicular cancer and anti-Ma2/Ta antibodies [20].

Such autoantibodies can be excellent markers for the diagnosis of neurological pathologies and underlying neoplasms. However, these antibodies do not seem to have a direct pathogenic role in observed neurological symptoms, as antibody removal therapy is not effective and immunization with the target intracellular proteins or patient antibodies cannot reproduce disease features. Interestingly, it has been shown that neuronal tissues from patients with limbic encephalitis and anti-Hu antibodies were infiltrated by massive lymphocyte numbers, mostly comprised of CD8+ T cells. Further, CD8+T cells among peripheral mononuclear cells obtained from PNS patients with anti-Yo or anti–Hu antibodies had cytotoxic activity against on neurons expressing Yo or Hu antigens [21,22,23,24]. Additionally, anti-Yo- or anti-Hu-positive patients share common human leukocyte antigen (HLA) class I motifs within each group [25]. This indicates that, in patients with anti-Yo or anti-Hu antibodies, antigenic peptides can be presented on antigen-presenting cells, such as dendritic cells, to stimulate cognate CD8+T cells, which then attack target tissues as effector cells. Activated antigen-responsive T cells enter the CNS and directly damage relevant neurons, resulting in rapid and severe neurological disease with a poor prognosis. Young male patients with PNS associated with testicular cancer and anti-Ma2 antibodies are known to have exceptionally better disease prognoses [20].

### 2.2. Antibodies against Intracellular Synaptic Sites

Patients with stiff-person syndrome, progressive encephalomyelitis with rigidity and myoclonus (PERM), or cerebellar ataxia associated with breast cancer or small cell lung cancer (SCLC) have antibodies against one or more proteins of GABAergic or glycinergic synapses, including glutamic acid decarboxylase 65 (GAD65), the glycine receptor (GlyR), or amphiphysin [26,27,28,29].

Amphiphysin and GAD65 locate to intracellular synaptic sites and are usually difficult to recognize as target antigens. They are enriched in the presynaptic nerve terminal and have roles in endocytosis, which implies that these antigens may be transiently exposed to the extracellular space [30]. In general, IgG and other macromolecules can be taken up by cells in a nonspecific manner. Once taken up, antibodies may be degraded before finding their target antigen, and thus may not be pathogenic. However, there have been passive transfer experiments with IgG fractions from patients with stiff-person syndrome and anti-amphiphysin antibodies, revealing motor hyperactivity and stiffness in mice [31]. For GAD65 antibodies, the intrathecal application of patient IgG fractions containing GAD65 antibodies induced symptoms similar to those of donor patients, but these observations have not been confirmed [32].

### 2.3. Antibodies against Cell Surface Synaptic Antigens

Autoantibodies against cell surface receptors at the neuromuscular junction have been identified in myasthenia gravis [1,2] and Lambert–Eaton myasthenic syndrome (LEMS) with anti-voltage-gated calcium channel (VGCC) antibodies [33]. In CNS disorders, autoantibodies against cell surface proteins were first reported in two patients with limbic encephalitis without an underlying neoplasm and without previously characterized PNS-related antibodies. These patients had autoantibodies against cell surface proteins interacting with voltage-gated potassium channels (VGKC) [7]. In 2007, anti-NMDAR antibodies were discovered as being closely related to autoimmune encephalitis in young women with severe psychiatric symptoms, other characteristic symptoms, and teratomas [9,10]. After this case, several reports of autoantibodies against over 16 neuron and glial cell plasma membrane proteins relating to autoimmune encephalitis were published. The targets included leucine-rich glioma-inactivated 1 (LGI1), contactin-associated protein-like 2 (CASPR2) [34,35] (both constituents of the VGKC complex), the α-amino-3-hydroxy-5-methyl-4-isoxazolepropionate receptor (AMPAR) [36,37,38], GABA B and A receptors (GABA_B_R, GABA_A_R) [39,40,41], the glycine receptor (GlyR) [42], dipeptidyl peptidase-like protein 6 (DPPX6) [43], metabotropic glutamate receptors 1 and 5 (mGlyR1 and mGluR5) [44,45], and others. Among these autoantibodies, anti-NMDAR antibodies are the most frequently detected in autoimmune encephalitis [46,47]. Anti-LGI1 antibodies are the second most detected autoimmune encephalitis-related antibodies, usually found in older patients (mostly in their 60s) suffering from memory disturbance, seizures, and other symptoms of limbic encephalitis [48].

Recently, extensive antibody tests have been performed and have revealed some patients with multiple antibodies in their sera/CSF. In these cases, the combination of anti-NMDAR antibodies and antibodies related to demyelinating disease, such as anti-AQP4 antibodies and anti-MOG antibodies, is frequently reported [49,50,51,52]. Most of these patients have clinical features related to each antibody throughout the disease course. For example, a patient with anti-NMDAR antibodies and anti-MOG antibodies will suffer from an aggressive mood disorder frequently seen in anti-NMDAR encephalitis, followed by optic neuritis and cerebral cortex lesions revealed by brain MRI, a clinical feature of anti-MOG antibody-related disease. These three antigens are all present in the CNS, however at different locations. NMDARs are present in neuronal synapses and AQP4 is located at the astrocyte endfeet in contact with vessels, while MOG is located on the surface of myelin or oligodendrocytes. The triggers of these pathological combinations are not known. None of the three antibodies are produced as a secondary phenomenon of neuronal tissue breakdown. These patients may have some immunological predisposition that causes the reaction to multiple antigens.

## 3. Anti-NMDAR Encephalitis

Anti-NMDAR encephalitis had been categorized as PNS associated with ovarian teratoma [9]. However, more than half of the cases are not associated with a tumor and are now believed to occur as primary autoimmune disease. Anti-NMDAR encephalitis mostly affects previously healthy young women (median age: 21 years; range: 8 months to 85 years; extreme prevalence of female cases (80%)), however 40% of patients aged under 12 or over 45 years are male and develop unique clinical characteristics. The initial symptoms are of acute psychiatric nature and include hallucinations, paranoid thoughts, insomnia, aggressive behavior, and catatonia, bringing patients to the psychiatric ward. Reports from psychiatrists state that the frequent early symptoms of anti-NMDAR encephalitis are behavioral changes, psychosis, mood swings, catatonia, and sleep disturbance [53,54]. The most frequent symptom combinations for suspected encephalitis in the early stage are mood changes and psychosis, especially in previously healthy young women. Following or together with these psychiatric symptoms, patients show disorders of consciousness, seizures, respiratory failure, bizarre involuntary movements, and autonomic disturbances, including unstable blood pressure, cardiac arrhythmia, hypersalivation, ileus, and respiratory failure. Patients with anti-NMDAR encephalitis usually have to be treated under sedation in an intensive care unit for several weeks to months. Some patients experience a prodromal viral infection with flu-like symptoms. A small number of patients were reported to develop anti-NMDAR encephalitis after herpes simplex virus encephalitis (HSE), previously termed as “relapsing neurologic symptoms post-HSE”, and responded to immunological treatments [55,56]. Almost 20% of HSE patients are reported to develop antibodies against neuronal cell surface proteins, mainly anti-NMDAR antibodies, suggesting that prodromal CNS viral infections might be related to autoimmune encephalitis [57].

Common laboratory tests are not specific for anti-NMDAR encephalitis. The patient CSF tends to show mild pleocytosis with elevated protein content, but some cases have normal results. Oligoclonal IgG bands in the CSF are observed in two thirds of patients. An MRI of the brain reveals abnormalities in the hippocampal area, cortex, subcortex, brainstem, or cerebellum in less than 30% of the patients. Diffused slow waves are most frequently observed in electroencephalograms even in patients with frequent seizures, and 23% of patients show epileptic spikes and sharp waves, especially at the early stage [58].

Patients are treated with corticosteroids, intravenous immunoglobulin, or plasmapheresis as first-line immunotherapy along with tumor resection, if applicable. If the treatment response is poor, rituximab or cyclophosphamide can be used. Titulae et al. reported that out of 501 NMDAR encephalitis patients treated using such a course of treatment, 81% improved to the level of “no symptoms” or “slight disability” during the first 24 months [46]. However, 9.5% died, and anti-NMDAR encephalitis recurred in 12%. It has been generally expressed that patients with this disease experience favorable recovery. However, a certain number of patients have prolonged personality changes and memory disturbances.

**Figure 1:**
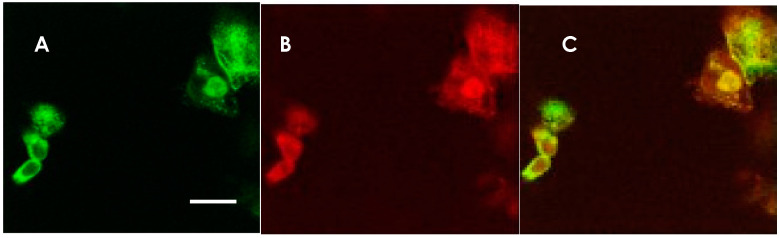
Anti-NMDAR antibody-detection using NMDAR GluN1 and GluN2B co-transfected HEK 293 cells. Immunostaining of HEK 293 cells expressing NMDARs in a patient’s CSF and a mixture of rabbit anti-NMDAR antibodies. The same cells were doubly stained with a patient’s CSF and a mixture of rabbit anti-GluN1 and anti-GluN2B antibodies. (**A**) Staining with CSF from an anti-NMDAR encephalitis patient. AlexaFluor 488-conjugated anti human IgG was used for the secondary antibody (green). (**B**) Staining with rabbit anti-GluN1 and anti-GluN2B antibody mixture. AlexaFluor 594-conjugated secondary antibody was used (red). (**C**) Superimposition of the two micrographic images, indicating that the CSF was positive in the anti-NMDAR antibodies. The scale bar in A (10 μm) applies also to B and C.

The most reliable diagnostic approach for anti-NMDAR encephalitis is based on the detection of anti-NMDAR IgG antibodies in the CSF. In our lab, we have tested for anti-NMDAR antibodies using GluN1- and GluN2B-co-transfected HEK-293 cells as antigens, and the bound antibodies were detected through immunofluorescence staining (Figure 1). Autoantibody titers usually correlate with disease activity.

The IgG isotype is believed to be important for the pathogenesis of anti-NMDAR encephalitis. There have been reports stating that certain psychotic patients (with schizophrenia, mood disorders, etc.) have IgM or IgA anti-NMDAR antibodies, usually present in low titers in patient sera [59,60]. Pruss et al. reported seven patients with IgA antibodies against NMDAR in their sera. These anti-NMDAR IgA antibodies in serum caused a dramatic decrease in the levels of NMDAR and other synaptic proteins in cultured neurons, along with prominent changes in NMDAR-mediated currents. These effects correlated with the titer of anti-NMDAR IgA antibodies and were reversed after removing the patient sera from the culture media. Further, comprehensive clinical assessments and brain metabolic imaging revealed neurologic improvement after immunotherapy and concluded that a subset of patients with slowly progressive cognitive impairment had underlying synaptic autoimmunity with anti-NMDAR IgA antibodies [61].

Considering the relationship between autoantibodies and encephalitic inflammation, antibody-mediated autoimmune encephalitis should be abruptly caused by the production of antigen-specific, high-affinity IgG antibodies, whereas naturally occurring IgA antibodies might chronically affect the maturation of synapses, in turn causing neuropsychiatric disturbances [62].

### Role of Antibodies in Anti-NMDAR Encephalitis

About 30–40% of anti-NMDAR encephalitis cases are associated with teratomas expressing neuronal antigens inside the tumor that might sensitize peripheral lymphocytes. Anti-NMDAR antibodies were shown to bind to neurons in the hippocampus; however, no neuronal death or deposits of complement were observed, even though the autoantibodies were predominantly IgG1, a subclass that can activate complement [63]. Anti-NMDAR antibodies recognize neuronal cell surface NMDARs in their native conformation. Thus, antibody detection requires the use of native antigens expressed on the cell surface. Such detection can be achieved through the immunohistochemistry of CNS tissue, cultured neuronal cells, or relevant cDNA-transfected cultured cells (usually using HEK293 cells) as sources of antigen. NMDARs are comprised of four subunits. GluN1 is expressed together with GluN2 or GluN3, and these complexes are usually observed as a combination of two GluN1 subunits and two GluN2 subunits (GluN2A or GluN2B) [64,65]. The most important site of antibody binding is located at the N368/G369 region of the extracellular domain. Interestingly, patient antibodies could not react to small peptides containing this region [66]. Antibody detection is now achievable through commercial detection kit systems using GluN1-mono-transfected HEK293 cells and called anti-NR1 antibodies.

The role of anti-NMDAR antibodies has been extensively investigated. Striking symptom similarities have been observed in models treated with NMDAR-specific antagonists, such as phencyclidine and ketamine [67]. In patients with anti-NMDAR-encephalitis, antibody removal through plasmapheresis and the suppression of antibody production through immunosuppressive therapy ameliorate the disease. These observations have incentivized studies with the aim to determine whether autoantibodies are pathogenic. Initial studies using dissociated rat hippocampal neurons incubated with patient antibodies for 3–7 days showed a selective and reversible decrease in NMDAR surface density, and a whole-cell patch-clamp procedure of cultured neurons revealed that patient autoantibodies specifically decreased the synaptic NMDAR-mediated currents without altering the AMPA receptor-mediated currents [63].

We showed that the IgG from patients with anti-NMDAR encephalitis inhibited the induction of long-term potentiation (LTP) in mouse hippocampal slices. The inhibition of LTP induction was reversed through treatment with antibody-depleted patient CSF. Moreover, the inhibition of LTP induction could not be detected after treatment with CSF samples from viral meningoencephalitis, neuroinflammatory disorders such as multiple sclerosis, or other neurodegenerative diseases, suggesting that the anti-NMDAR antibodies in patient CSF are closely related to the memory disturbance observed in anti-NMDAR encephalitis patients [68].

To further understand the pathological mechanisms of this disease, we examined the effects of autoantibodies on the behavior of mice that were injected (in the lateral ventricles) with the IgG fraction of the CSF from anti-NMDAR-Ab-positive patients. We also assessed the histological alterations in the brain tissue of these mice. The IgG from patients’ CSF positive for anti-NMDAR antibodies was infused into the lateral ventricles of mice continuously for 4 weeks using an osmotic pump. Serial behavior tests such as spontaneous locomotor activity, the open field test, the novel object recognition test, and the Morris water maze test were then performed. Mice treated with NMDAR-CSF demonstrated the deterioration of spatial memory functions, as assessed by the Morris water maze test. The autoantibodies were predominantly IgG1; however, no complement deposition was observed in the brain tissue [69].

Planagumà et al. also reported that the mice treated with anti-NMDAR encephalitis patients’ CSF infused into both lateral ventricles showed memory disturbance, as revealed by an object recognition test. The mice recovered after CSF infusions were discontinued [70]. The observed effects occurred in parallel to anti-NMDAR IgG binding to the mouse brain, as revealed through the bound IgG extraction methods. This study and our work confirm that patient anti-NMDAR antibodies directly cause the memory disturbance that is observed in patients suffering from anti-NMDAR encephalitis. However, the continuous infusion of a large volume of antibody-positive CSF could not reproduce the wide range of remaining symptoms.

Taraschenko et al. reported that patient CSF or purified IgG induced frequent seizures in 33 of 36 mice. Memory deficits, anxiety-related behavior, or motor impairment were not observed upon assessment after 2 weeks of CSF treatment. Furthermore, there was no evidence of hippocampal cell loss or astrocyte proliferation [71]. Taken together, the available evidence has not yet fully confirmed the pathogenic role of anti-NMDAR antibodies in autoimmune encephalitis.

## 4. CNS Diseases Associated with Cell Surface-Targeting Antibodies Other Than Anti-Nmdar Antibodies

Autoantibodies that have been associated with encephalitis include antibodies against over 16 targets. Among them, antibodies targeting the AMPA receptor (AMPAR), GABA_B_ receptor, and LGI1 are related to the symptoms of limbic encephalitis. The LGI1, GABA_B_R, and AMPAR pathologies may have a more indolent course causing confusion, behavioral changes, seizures, and memory disturbance, and tend occur in older patients. They do not show extreme female predominance, as with anti-NMDAR encephalitis.

### 4.1. Anti-AMPAR Antibodies

The AMPAR is an ionotropic glutamate receptor present as the tetramers of GluA1/2 and GluA2/3. Patients with anti-AMPAR antibodies are usually older than those with anti-NMDAR antibodies, and mainly have the symptoms of limbic encephalitis. About 70% of them have an underlying tumor, such as SCLC or thymoma. Immunotherapy or tumor treatment are effective for their neurological symptoms, but some experience relapses [38].

### 4.2. Anti-LGI1/Anti-CASPR2 Antibodies

The autoantibodies formerly referred to as anti-VGKC antibodies are now termed anti-LGI1 or anti-Caspr2 antibodies, depending on their direct target antigen [33,34]. LGI1 is a secreted glycoprotein that interacts with presynaptic ADAM (a disintegrin and metalloprotease) 23 and postsynaptic ADAM22, organizing a trans-synaptic protein complex which includes presynaptic Kv1.1 potassium channels and postsynaptic AMPA receptors [72,73]. The LGI1 protein was initially found in the dentate molecular layer and granule cell mossy fibers, and it was thought to be secreted from both axonal presynapses and dendritic postsynapses [74]. The mutations of *LGI1* cause autosomal dominant partial epilepsy with auditory seizures (autosomal dominant lateral temporal lobe epilepsy: ADLTE) [75]. LGI1 knockout in mice or the preincubation of primary neurons with patient anti-LGI1 antibodies induces the downregulation of synaptic AMPARs; however, there is no direct evidence of LGI1 antibody-mediated effects on neuronal excitability and synaptic transmission [76].

Anti-LGI1 antibody-positive patients are usually of an older age (median age: 60 years); there is a slight male predominance, and 60% of the patients have hyponatremia. The symptoms of limbic dysfunction can be preceded by faciobrachial dystonic seizures that last a few seconds and may occur many times during the day [77]. MRI reveals basal ganglia hyperintensity in these patients. About 70% of patients improve after immunotherapy, but over 70% of them show residual cognitive dysfunction. Most patients with anti-LGI1 antibodies do not have cancer. Anti-LGI1 antibodies and anti-CASPR2 antibodies are mainly IgG4 and do not fix complement, which differs from other antibodies related to limbic encephalitis [48].

Patients with anti-CASPR2 antibodies develop limbic encephalitis sometimes associated with neuromyotonia and autonomic symptoms (Morvan syndrome). Approximately 20% of the patients also have a thymoma. Immunotherapy and tumor treatment results in an improvement in 93% of the patients, while 25% of patients experience relapses [78].

### 4.3. Anti-GABA_B_R Antibodies

GABA_B_R is a G protein-coupled receptor for the inhibitory neurotransmitter GABA. Patients with autoantibodies against this receptor have clinical features of limbic encephalitis associated with seizures (status epilepticus). Approximately 50% of the patients have SCLC. Most patients show favorable outcomes with immunotherapy and tumor treatment; however, refractory status epilepticus could occur [40].

### 4.4. Anti-GABA_A_R Antibodies

The GABA_A_R is a ligand-gated ion channel that mediates the majority of fast inhibitory transmission in the brain. GABA_A_Rs are heteropentamers consisting of five homologous subunits; most of them contain two α, two β, and one γ or δ subunit. In patients with autoantibodies against the GABA_A_R, the predominant targets are subunits α1 and β3 [76]. Patients with GABA_A_R antibodies are characterized by psychiatric disorders, cognitive deficits, prominent seizures, or status epilepticus. MRI shows multifocal T2/FLAIR high-signal lesions in the cerebrum [79].

### 4.5. Anti-mGluR5 Antibodies

Eight subtypes of the metabotropic glutamate receptor (mGluR) are known in mammals. Among them, mGluR1 and mGluR5 are reported to be targets in autoimmune encephalitis. Patients with anti-mGluR1 antibodies show cerebellar ataxia, while limbic encephalitis occurs as an anti-mGluR5 antibody-related disorder. mGluR5 regulates rapid synaptic transmission in the hippocampus via its functional interaction with NMDAR in LTP regulation [80]. Patients with anti-mGluR5 antibodies develop psychiatric symptoms associated with limbic encephalitis. This pathology is frequently associated with Hodgkin’s lymphoma; however, the neurological symptoms are quickly alleviated with immunotherapy [45].

### 4.6. Anti-GlyR Antibodies

GlyR is a postsynaptic chloride channel receptor mainly expressed in the brain stem and spinal cord [81]. It is involved in inhibitory synaptic transmission and the fine regulation of motor neuron excitability. Anti-GlyR α-subunit antibodies are associated with muscle stiffness and painful spasms in the trunk and extremities that are easily triggered by light or emotional stimuli. The symptoms include seizures and dysautonomia (stiff-person syndrome), as well as PERM. Limbic and brain stem encephalitis with opisthotonus, hypersomnia, neuropathic pain, and pruritus are also observed [42,82]. The symptoms improve during sleep and through the administration of diazepam and other GABAergic drugs. Electrophysiological examinations have revealed sustained the co-contraction of agonist and antagonist muscles, and these findings are important for the diagnosis of this disease [83].

## 5. Conclusions

The recent discoveries of several autoantibodies produced in patients with autoimmune encephalomyelitis have expanded new clinical entities, such as autoimmune psychosis and autoimmune epilepsy, and also provide a deep understanding of the background of neurological symptoms in these disorders, together with new insights into the basic neuroscience. For patients with psychotic disorders or intractable epilepsy previously treated in psychiatric wards, there is a possibility for effective treatment through immunotherapy, such as intravenous methylprednisolone infusion, high-dose immunoglobulin administration, plasmapheresis, or other immunosuppressants. Synaptic receptor dysfunction due to antibody binding causes various neurological symptoms of encephalopathy-associated pathologies. It is unclear why autoantibodies target such proteins, which are widely expressed in the brain, or why different pathophysiological mechanisms resulting from different targeted synaptic proteins converge into a similar syndrome. Each clinical feature might be the consequence of alterations in a synaptic signaling process. However, the experimental evidence describing these relationships is still very poor. Investigations into the mechanisms underlying these phenomena might bring further insight into the altered signal transduction that takes place in CNS networks. In addition, the growing body of knowledge on immunological alterations in the peripheral immune system and CNS inflammation resulting from such autoimmune diseases may provide hints for the relationship between neurodegeneration and neuroinflammation in the future.

## Figures and Tables

**Table 1:** Autoantibody targets and associated syndromes (modified from [14]).

Intracellular Antigens			
Antigen	Syndromes	Tumor Association	Mechanisms
Hu (HuD)	limbic encephalitis encephalomyelitis cerebellar ataxia sensory neuronopathy autonomic neuropathy	SCLC	CTL
Yo (CDR2/CDR62)	cerebellar ataxia	ovary, uterus breast	CTL
Ma1/2 (MA)	limbic/brainstem encephalitis	germ-cell tumors of testis	unclear
CRMP5	encephalomyelitis polyneuropathy cerebellar ataxia	SCLLC thymoma	CTL
Tr (DNER)	cerebellar ataxia	lymphoma	unclear
Ri (NOVA-1)	opsoclonus-nyoclonus rhomboencephalitis cerebellar ataxia	breast, ovary SCLC	unclear
Recoverin	retinopathy	SCLC	unclear
**Intracellular synaptic antigens**			
GAD65	Stiff-person syndrome cerebellar ataxia	thymoma	unclear
Amphiphysin	Stiff-person syndrome Limbic encephalitis cerebellar ataxia polyneuropathy	breast SCLC	Ab
**Extracellular/cell membrane**			
NMDAR	encephalitis	teratoma	Ab
AMPAR	limbic encephalitis	lung, breast thymic cancer	Ab
LGI1	limbic encephalitis	lung, thymoma	unclear
CASPR2	encephalitis (Morvan syndrome) neuromyotonia	lung, thymoma	unclear
GABA_B_R	limbic encephalitis prominent seizures	SCLC	Ab
GABA_A_R	encephalitis status epileptics	thymoma	Ab
mGluR1	cerebellar ataxia	Hodgkin lymphoma	Ab
GlyR	PERM	thymoma	Ab
VGCC	LEMS cerebellar ataxia	SCLC	Ab
mGluR5	limbic encephalitis	Hodgkin lymphoma	Ab

SCLC: small cell lumg cancer; CTL: cytotoxic T lymphocyte; CRMP5: collapsing response mediator protein 5; DNER: delta/notch-like epidermal growth factor-related receptor; Ab: antibody-mediated disturbance of receptor/channel function; NMDAR: *N*-methyl-d-aspartate; AMPAR: α-amino-3-hydroxy-5-methyl-4-isoxazolepropionic receptor; LGI1: leucine-rich glioma inactivated 1; CASPR2: contactine-associated protein-like 2; GABA_B_R: γ-aminobutyric acid type B receptor; GABA_A_R: γ-aminobutyric acid type A receptor; mGluR1: metabotropic glutamate receptor 1; GlyR: glycine receptor; VGCC: voltage-gated calcium channel; LEMS: Lambert–Eaton myasthenic syndrome; mGluR5: metabotropic glutamate receptor 5.

## Abbreviations

CNS	Central nervous system
AEM	Autoimmune ecncephalomyelitis
CSF	Cerebrospinal fluid
AQP4	Aquaporin 4
PNS	Paraneoplastic neurological syndrome
HLA	Human leukocyte antigen
PERM	Progressive encephalomyelitis with rigidity and myoclonus
SCLC	Small cell lung cancer
GAD	Glutamic acid decarboxylase
GlyR	Glycine receptor
VGKC	Voltage-gated potassium channel
NMDAR	N-methyl-D-aspafrtate receptor
CTL	Cytotoxic T lymphocyte
CRMP5	Collapsing response mediator protein 5
DNER	Delta/notch-like epidermal growth factor-related receptor
Ab	antibody-mediated disturbance of receptor/channel function
AMPAR	α-amino-3-hydroxy-5-methyl-4-isoxazolepropionic receptor
LGI1	leucine-rich glioma inactivated 1
CASPR2	contactine-associated protein-like 2
GABA_B_R	γ-aminobutyric acid type B receptor
GABA_A_R	γ-aminobutyric acid type A receptor
VGCC	voltage-gated calcium channel
LEMS	Lambert-Eaton myasthenic syndrome
mGluR1/5	metabotropic glutamate receptor 1/5

## References

[1] Drachman D.B., Adams R.N., Josifek L.F., Self S.G. (1982). Functional activities of autoantibodies to acetylcholine receptors and the clinical severity of myasthenia gravis. N. Engl. J. Med..

[2] Drachman D.B., Angus C.W., Adams R.N., Michelson J.D., Hoffman G.J. (1978). Myasthenic antibodies cross-link acetylcholine receptors to accelerate degradation. N. Engl. J. Med..

[3] Darnell R.B., Posner J.B. (2003). Paraneoplastic syndromes involving the nervous system. N. Engl. J. Med..

[4] Dalmau J., Rosenfeld M.R. (2008). Paraneoplastic syndromes of the CNS. Lancet Neurol..

[5] Höftberger R., Rosenfeld M.R., Dalmau J. (2015). Update on neurological paraneoplastic syndromes. Curr. Opin. Oncol..

[6] Leypoldt F., Wandinger K.P. (2014). Paraneoplastic neurological syndromes. Clin. Exp. Immunol..

[7] Buckley C., Oger J., Clover L., Tuzun E., Carpenter K., Jackson M., Vincent A. (2001). Potassium channel antibodies in two patients with reversible limbic encephalitis. Ann. Neurol..

[8] Lennon V.A., Kryzer T.J., Pittock S.J., Verkman A.S., Hinson S.R. (2005). IgG marker of optic-spinal multiple sclerosis binds to the aquaporin-4 water channel. J. Exp. Med..

[9] Dalmau J., Tüzün E., Wu H., Masjuan J., Rossi J.E., Voloschin A., Baehring J.M., Shimazaki H., Koide R., King D. (2007). Paraneoplastic Anti–*N*-methyl-d-aspartate receptor encephalitis associated with ovarian teratoma. Ann. Neurol..

[10] Dalmau J., Gleichman A.J., Hughes E.G., Rossi J.E., Peng X., Lai M., Dessain S.K., Rosenfeld M.R., Balice-Gordon R., Lynch D.R. (2008). Anti-NMDA-receptor encephalitis: Case series and analysis of the effects of antibodies. Lancet Neurol..

[11] Dalmau J., Graus F. (2018). Antibody-Mediated encephalitis. N. Engl. J. Med..

[12] Ramanathan S., Mohammad S., Tantsis E., Nguyen T.K., Merheb V., Fung V.S.C., White O.B., Broadley S., Lechner-Scott J., Vucic S. (2018). Clinical course, therapeutic responses and outcomes in relapsing MOG antibody-associated demyelination. J. Neurol. Neurosurg. Psychiatry.

[13] Rose N.R., Bona C. (1993). Defining criteria for autoimmune diseases (Witebsky’s postulates revisited). Immunol. Today.

[14] Tanaka K. (2019). Autoimmune encephalomyelitis. Clin. Exp. Neuroimmunol..

[15] Bernal F., Graus F., Pifarre A., Saiz A., Benyahia B., Ribalta T. (2002). Immunohistochemical analysis of anti-Hu-associated paraneoplastic encephalomyelitis. Acta Neuropathol..

[16] Alamowitch S., Graus F., Uchuya M., Rene R., Bescansa E., Delattre J.Y. (1997). Limbic encephalitis and small cell lung cancer. Clinical and immunological features. Brain.

[17] Rojas I., Graus F., Keime-Guibert F., Rene R., Delattre J.Y., Ramon J.M., Dalmau J., Posner J.B. (2000). Long-Term clinical outcome of paraneoplastic cerebellar degeneration and anti-Yo antibodies. Neurology.

[18] Weizman D.A., Leong W.L. (2004). Anti-Ri antibody opsoclonus-myoclonus syndrome and breast cancer: A case report and a review of the literature. J. Surg. Oncol..

[19] Honnorat J., Antoine J.C., Belin M.F. (2001). Are the “newly discovered” paraneoplastic anticollapsin response-mediator protein 5 antibodies simply anti-CV2 antibodies?. Ann. Neurol..

[20] Dalmau J., Graus F., Villarejo A., Posner J.B., Blumenthal D., Thiessen B., Saiz A., Meneses P., Rosenfeld M.R. (2004). Clinical analysis of anti-Ma2-associated encephalitis. Brain.

[21] Tanaka M., Tanaka K. (2002). Cytotoxic T cell activity against peptides of Hu protein in anti-Hu syndrome. J. Neurol. Sci..

[22] Tanaka K., Tanaka M., Onodera O., Igarashi S., Miyatake T., Tsuji S. (1994). Passive transfer and active immunization with the recombinant leucine-zipper (Yo) protein as an attempt to establish an animal model of paraneoplastic derebellar degeneration. J. Neurol. Sci..

[23] Tanaka M., Tanaka K., Onodera O., Tsuji S. (1995). Trial to establish an animal model of paraneoplastic cerebellar degeneration (PCD) with anti-Yo antibody. 1. Mice strains bearing different MHC molecules produced antibodies on immunization with recombinant Yo protein, a T-dependent antigen, but do not cause Purkinje cell loss. Clin. Neurol. Neurosurg..

[24] Albert M.L., Darnell J.C., Bender A., Francisco L.M., Bhardwaj N., Darnell R.B. (1998). Tumor-specific killer cells in paraneoplastic cerebellar degeneration. Nat. Med..

[25] Tanaka M., Tanaka K., Tsuji S., Kawata A., Kojima S., Kurokawa T., Kira J., Takiguchi M. (2001). Cytotoxic T cell activity against the peptide, AYRARALEL, from Yo protein of patients with the HLA A24 or B27 supertype and paraneoplastic cerebellar degeneration. J. Neurol. Sci..

[26] Pittock S.J., Lucchinetti C.F., Parisi J.E., Benarroch E.E., Mokri B., Stephan C.L., Kim K.K., Kilimann M.W., Lennon V.A. (2005). Amphiphysin autoimmunity: Paraneoplastic accompaniments. Ann. Neurol..

[27] Solimena M., Folli F., Aparisi R., Pozza G., De Camilli P. (1990). Autoantibodies to GABA-ergic neurons and pancreatic beta cells in stiff-man syndrome. N. Engl. J. Med..

[28] Arino H., Gresa-Arribas N., Blanco Y., Martínez-Hernández E., Sabater L., Petit-Pedrol M., Rouco I., Bataller L., Dalmau J.O., Saiz A. (2014). Cerebellar ataxia and glutamic acid decarboxylase antibodies: Immunologic profile and long-term effect of immunotherapy. JAMA Neurol..

[29] De Camilli P., Thomas A., Cofiell R., Folli F., Lichte B., Piccolo G., Meinck H.M., Austoni M., Fassetta G., Bottazzo G. (1993). The synaptic vesicle-associated protein amphiphysin is the 128-kD autoantigen of Stiff-Man syndrome with breast cancer. J. Exp. Med..

[30] Bauerfeind R., Takei K., De Camili P. (1997). Amphiphysin I is associated with coated endocytic intermediates and undergoes stimulation-dependent dephosphorylation in nerve terminals. J. Biol. Chem..

[31] Sommer C., Weishaupt A., Brinkhoff J., Biko L., Wessig C., Gold R., Toyka K.V. (2005). Paraneoplastic stiff-person syndrome: Passive transfer to rats by means of IgG antibodies to amphiphysin. Lancet.

[32] Hansen N., Grunewald B., Weishaupt A., Colaco M.N., Toyka K.V., Sommer C., Geis C. (2013). Human Stiff person syndrome IgG-containing high-titer anti-GAD65 autoantibodies induce motor dysfunction in rats. Exp. Neurol..

[33] Waterman S.A., Lang B., Newsom-Davis J. (1997). Effect of Lambert-Eaton myasthenic syndrome antibodies on autonomic neurons in the mouse. Ann. Neurol..

[34] Irani S.R., Alexander S., Waters P., Kleopa K.A., Pettingill P., Zuliani L., Peles E., Buckley C., Lang B., Vincent A. (2010). Antibodies to Kv1 potassium channel-complex proteins leucine-rich, glioma inactivated 1 protein and contactin-associated protein-2 in limbic encephalitis, Morvan’s syndrome and acquired neuromyotonia. Brain.

[35] Lai M., Huijbers M.G., Lancaster E., Graus F., Bataller L., Balice-Gordon R., Cowell J.K., Dalmau J. (2010). Investigation of LGI1 as the antigen in limbic encephalitis previously attributed to potassium channels: A case series. Lancet Neurol..

[36] Lai M., Hughes E.G., Peng X., Zhou L., Gleichman A.J., Shu H., Matà S., Kremens D., Vitaliani R., Geschwind M.D. (2009). AMPA receptor antibodies in limbic encephalitis alter synaptic receptor location. Ann. Neurol..

[37] Bataller L., Galiano R., García-Escrig M., Martínez B., Sevilla T., Blasco R., Vílchez J.J., Dalmau J. (2010). Reversible paraneoplastic limbic encephalitis associated with antibodies to the AMPA receptor. Neurology.

[38] Hoftberger R., van Sonderen A., Leypoldt F., Houghton D., Geschwind M., Gelfand J., Paredes M., Sabater L., Saiz A., Titulaer M.J. (2015). Encephalitis and AMPA receptor antibodies: Novel findings in a case series of 22 patients. Neurology.

[39] Höftberger R., Titulaer M.J., Sabater L., Dome B., Rózsás A., Hegedus B., Hoda M.A., Laszlo V., Ankersmit H.J., Harms L. (2013). Encephalitis and GABAB receptor antibodies: Novel findings in a new case series of 20 patients. Neurology.

[40] Lancaster E., Lai M., Peng X., Hughes E., Constantinescu R., Raizer J., Friedman D., Skeen M.B., Grisold W., Kimura A. (2010). Antibodies to the GABA(B) receptor in limbic encephalitis with seizures: Case series and characterization of the antigen. Lancet Neurol..

[41] Pettingill P., Kramer H.B., Coebergh J.A., Pettingill R., Maxwell S., Nibber A., Malaspina A., Jacob A., Irani S.R., Buckley C. (2015). Antibodies to GABAA receptor alpha 1 and gamma 2 subunits clinical and serologic characterization. Neurology.

[42] Carvajal-Gonzalez A., Leite M.I., Waters P., Woodhall M., Coutinho E., Balint B., Lang B., Pettingill P., Carr A., Sheerin U.-M. (2014). Glycine receptor antibodies in PERM and related syndromes: Characteristics, clinical features and outcomes. Brain.

[43] Tobin W.O., Lennon V.A., Komorowski L., Probst C., Clardy S.L., Aksamit A.J., Appendino J.P., Lucchinetti C.F., Matsumoto J.Y., Pittock S.J. (2014). DPPX potassium channel antibody: Frequency, clinical accompaniments, and outcomes in 20 patients. Neurology.

[44] Smitt P.S., Kinoshita A., De Leeuw B., Moll W., Coesmans M., Jaarsma D., Henzen-Logmans S., Vecht C., De Zeeuw C., Sekiyama N. (2000). Paraneoplastic cerebellar ataxia due to autoantibodies against a glutamate receptor. N. Engl. J. Med..

[45] Lancaster E., Martinez-Hernandez E., Titulaer M.J., Boulos M., Weaver S., Antoine J.-C., Liebers E., Kornblum C., Bien C.G., Honnorat J. (2011). Antibodies to metabotropic glutamate receptor 5 in the Ophelia syndrome. Neurology.

[46] Titulaer M.J., McCracken L., Gabilondo I., Armangue T., Glaser C., Iizuka T., Honig L.S., Benseler S.M., Kawachi I., Martinez-Hernandez E. (2013). Treatment and prognostic factors for long-term outcome in patients with anti-NMDA receptor encephalitis: An observational cohort study. Lancet Neurol..

[47] Dalmau J., Lancaster E., Martinez-Hernandez E., Rosenfeld M.R., Balice-Gordon R. (2011). Clinical experience and laboratory investigations in patients with anti-NMDAR encephalitis. Lancet Neurol..

[48] Ariño H., Armangué T., Petit-Pedrol M., Sabater L., Martinez-Hernandez E., Hara M., Lancaster E., Saiz A., Dalmau J., Graus F. (2016). Anti-LGI1-associated cognitive impairment: Presentation and long-term outcome. Neurology.

[49] Titulaer M., Höftberger R., Iizuka T., Leypoldt F., McCracken L., Cellucci T., Benson L.A., Shu H., Irioka T., Hirano M. (2014). Overlapping demyelinating syndromes and anti-NMDA receptor encephalitis. Ann. Neurol..

[50] Jarius S., Ruprecht K., Kleiter I., Borisow N., Asgari N., Pitarokoili K., Pache F., Stich O., Beume L.-A., Hümmert M. (2016). MOG-IgG in NMO and related disorders: A multicenter study of 50 patients. Part 1: Frequency, syndrome specificity, influence of disease activity, long-term course, association with AQP4-IgG, and origin. J. Neuroinflamm..

[51] Di Pauli F., Mader S., Rostásy K., Schanda K., Bajer-Kornek B., Ehling R., Deisenhammer F., Reindl M., Berger T. (2011). Temporal dynamics of anti-MOG antibodies in CNS demyelinating diseases. Clin. Immunol..

[52] Tanaka M., Tanaka K. (2014). Anti-MOG antibodies in adult patients with demyelinating disorders of the central nervous system. J. Neuroimmunol..

[53] Al-Diwani A., Handel A., Townsend L., Pollak T., Leite M.I., Harrison P.J., Lennox B.R., Okai D., Manohar S.G., Irani S.R. (2019). The psychopathology of NMDAR-antibody encephalitis in adults: A systematic review and phenotypic analysis of individual patient data. Lancet Psychiatry.

[54] Kawai H., Takaki M., Sakamoto S., Shibata T., Tsuchida A., Yoshimura B., Yada Y., Matsumoto N., Sato K., Abe K. (2019). Anti-NMDA-receptor antibody in initial diagnosis of mood disorder. Eur. Neuropsychopharmacol..

[55] De Tiege X., Rozenberg F., Des Portes V., Lobut J.B., Lebon P., Ponsot G., Heron B. (2003). Herpes simplex encephalitis relapses in children: Differentiation of two neurologic entities. Neurology.

[56] Mohammad S.S., Sinclair K., Pillai S., Merheb V., Aumann T.D., Gill D., Dale R.C., Brilot F. (2014). Herpes simplex encephalitis relapse with chorea is associated with autoantibodies to N-methyl-d-aspartate receptor or dopamine-2 receptor. Mov. Disord..

[57] Armangue T., Moris G., Cantarin-Extremera V., Conde C.E., Rostasy K., Erro M.E., Portilla-Cuenca J.C., Turon-Vinas E., Malaga I., Munoz-Cabello B. (2015). Autoimmune post-herpes simplex encephalitis of adults and teenagers. Neurology.

[58] Schmitt S.E., Pargeon K., Frechette E.S., Hirsch L.J., Dalmau J., Friedman D. (2012). Extreme delta brush: A unique EEG pattern in adults with anti-NMDA receptor encephalitis. Neurology.

[59] Steiner J., Walter M., Glanz W., Sarnyai Z., Bernstein H.G., Vielhaber S., Kastner A., Skalej M., Jordan W., Schiltz K. (2013). Increased prevalence of diverse *N*-methyl-d-aspartate glutamate receptor antibodies in patients with an initial diagnosis of schizophrenia: Specific relevance of IgG NR1a antibodies for distinction from *N*-methyl-d-aspartate glutamate receptor encephalitis. JAMA Psychiatry.

[60] Hammer C., Stepniak B., Schneider A., Papiol S., Tantra M., Begemann M., Siren A.L., Pardo L.A., Sperling S., Mohd J.S. (2014). Neuropsychiatric disease relevance of circulating anti-NMDA receptor autoantibodies depends on blood-brain barrier integrity. Mol. Psychiatry.

[61] Prüss H., Höltje M., Maier N., Gomez A., Buchert R., Harms L., Ahnert-Hilger G., Schmitz D., Terborg C., Kopp U. (2012). IgA NMDA receptor antibodies are markers of synaptic immunity in slow cognitive impairment. Neurology.

[62] Tanaka K. (2018). Are naturally occurring anti-NMDAR autoantibodies pathogenic?. Nat. Rev. Neurol..

[63] Hughes E.G., Peng X., Gleichman A.J., Lai M., Zhou L., Tsou R., Parsons T.D., Lynch D.R., Dalmau J., Balice-Gordon R.J. (2010). Cellular and synaptic mechanisms of anti-NMDA receptor encephalitis. J. Neurosci..

[64] Dingledine R., Borges K., Bowie D., Traynelis S.F. (1999). The glutamate receptor ion channels. Pharmacol. Rev..

[65] Abe M., Fukaya M., Yagi T., Mishina M., Watanabe M., Sakimura K. (2004). NMDA receptor GluRepsilon/NR2 subunits are essential for postsynaptic localization and protein stability of GluRzeta1/NR1 subunit. J. Neurosci..

[66] Gleichman A.J., Spruce L.A., Dalmau J., Seeholzer S.H., Lynch D.R. (2012). Anti-NMDA receptor encephalitis antibody binding is dependent on amino acid identity of a small region within the GluN1 amino terminal domain. J. Neurosci..

[67] Kraguljac N.V., Carle M., Frölich M.A., Tran S., Yassa M.A., White D.M., Reddy A., Lahti A.C. (2018). Mnemonic eiscrimination deficits in First-Episode psychosis and a ketamineModel suggests dentate gyrus pathology linked to N-Methyl-D-AspartateReceptor Hypofunction. Biol. Psychiatry Cogn. Neurosci. Neuroimaging.

[68] Zhang Q., Tanaka K., Sun P., Nakata M., Yamamoto R., Sakimura K., Matsui M., Kato N. (2012). Suppression of synaptic plasticity by cerebrospinal fluid from anti-NMDA receptor encephalitis patients. Neurobiol. Dis..

[69] Li Y., Tanaka K., Wang L., Ishigaki Y., Kato N. (2015). Induction of memory deficit in mice with chronic exposure to cerebrospinal fluid from patients with Anti-N-Methyl-D-Aspartate receptor encephalitis. Tohoku J. Exp. Med..

[70] Planaguma J., Leypoldt F., Mannara F., Gutierrez-Cuesta J., Martin-Garcia E., Aguilar E., Titulaer M.J., Petit-Pedrol M., Jain A., Balice-Gordon R. (2015). Human N-methyl d-aspartate receptor antibodies alter memory and behaviour in mice. Brain.

[71] Taraschenko D.R.O., Fox H.S., Pittock S.J., Zekeridou A., Gafurova M., Eldridge E., Liu J., Dravid S.M., Dingledine R. (2019). A mouse model of seizures in anti-N-methyl D-aspartate receptor encephalitis. Epilepsia.

[72] Fukata Y., Adesnik H., Iwanaga T., Bredt D.S., Nicoll R.A., Fukata M. (2006). Epilepsy-related ligand/receptor complex LGI1 and ADAM22 regulate synaptic transmission. Science.

[73] Fukata Y., Lovero K.L., Iwanaga T., Watanabe A., Yokoi N., Tabuchi K., Shigemoto R., Nicoll R.A., Fukata M. (2010). Disruption of LGI1-linked synaptic complex causes abnormal synaptic transmission and epilepsy. Proc. Natl. Acad. Sci. USA.

[74] Lovero K.L., Fukata Y., Granger A.J., Fukata M., Nicoll R.A. (2015). The LGI1-ADAM22 protein complex directs synapse maturation through regulation of PSD-95 function. Proc. Natl. Acad. Sci. USA.

[75] Morante-Redolat J.M., Gorostidi-Pagola A., Piquer-Sirerol S., Saenz A., Poza J.J., Galan J., Gesk S., Sarafidou T., Mautner V.F., Binelli S. (2002). Mutations in the LGI1/Epitempin gene on 10q24 cause autosomal dominant lateral temporal epilepsy. Hum. Mol. Genet..

[76] Ohkawa T., Fukata Y., Yamasaki M., Miyazaki T., Yokoi N., Takashima H., Watanabe M., Watanabe O., Fukata M. (2013). Autoantibodies to epilepsy-related LGI1 in limbic encephalitis neutralize LGI1-ADAM22 interaction and reduce synaptic AMPA receptors. J. Neurosci..

[77] Irani S.R., Michell A.W., Lang B., Pettingill P., Waters P., Johnson M.R., Schott J.M., Armstrong R.J., Zagami S., Bleasel A. (2011). Faciobrachial dystonic seizures precede Lgi1 antibody limbic encephalitis. Ann. Neurol..

[78] Van Sonderen A., Arino H., Petit-Pedrol M., Leypoldt F., Kortvelyessy P., Wandinger K.P., Lancaster E., Wirtz P.W., Schreurs M.W., Sillevis Smitt P.A. (2016). The clinical spectrum of Caspr2 antibody-associated disease. Neurology.

[79] Petit-Pedrol M., Armangue T., Peng X., Bataller L., Cellucci T., Davis R., McCracken L., Martinez-Hernandez E., Mason W.P., Kruer M.C. (2014). Encephalitis with refractory seizures, status epilepticus, and antibodies to the GABAA receptor: A case series, characterisation of the antigen, and analysis of the effects of antibodies. Lancet Neurol..

[80] Kwag J., Paulsen O. (2012). Gating of NMDA receptor-mediated hippocampal spike timing-dependent potentiation by mGluR5. Neuropharmacology.

[81] Lynch J.W. (2004). Molecular structure and function of the glycine receptor chloride channel. Physiol. Rev..

[82] Mas N., Saiz A., Leite M.I., Waters P., Baron M., Castano D., Sabater L., Vincent A., Graus F. (2011). Anti glycine-receptor encephalomyelitis with rigidity. J. Neurol. Neurosurg. Psychiatry.

[83] Rakocevic G., Floeter M.K. (2012). Autoimmune Stiff Person Syndrome and related myelopathies:understanding of electrophysiological and immunological processes. Muscle Nerve.

